# Cardiac electrophysiological imaging systems scalable for high-throughput drug testing

**DOI:** 10.1007/s00424-012-1149-0

**Published:** 2012-09-29

**Authors:** Peter Lee, Ken Wang, Christopher E. Woods, Ping Yan, Peter Kohl, Paul Ewart, Leslie M. Loew, Derek A. Terrar, Christian Bollensdorff

**Affiliations:** 1 Department of Physics, Clarendon Laboratory, University of Oxford, Parks Road, Oxford, OX1 3PU UK; 2 Department of Computer Science, University of Oxford, Wolfson Building, Parks Road, Oxford, OX1 3QD UK; 3 Department of Cardiology, University of California, San Francisco, 500 Parnassus Ave, MU East, Rm 434, San Francisco, CA 94143-1354 USA; 4 Richard D. Berlin Center for Cell Analysis and Modeling, University of Connecticut Health Center, 263 Farmington Avenue, Farmington, CT 06030-6406 USA; 5 The Heart Science Centre, National Heart and Lung Institute, Imperial College London, Harefield, Middlesex UB9 6JH UK; 6 Department of Pharmacology, University of Oxford, Mansfield Road, Oxford, OX1 3QT UK

**Keywords:** Voltage and calcium-sensitive dyes, High-speed imaging, Optical mapping, Drug testing, High-throughput, Fluorescence, Multi-parametric, Cardiac

## Abstract

Multi-parametric electrophysiological measurements using optical methods have become a highly valued standard in cardiac research. Most published optical mapping systems are expensive and complex. Although some applications demand high-cost components and complex designs, many can be tackled with simpler solutions. Here, we describe (1) a camera-based voltage and calcium imaging system using a single ‘economy’ electron-multiplying charge-coupled device camera and demonstrate the possibility of using a consumer camera for imaging calcium transients of the heart, and (2) a photodiode-based voltage and calcium high temporal resolution measurement system using single-element photodiodes and an optical fibre. High-throughput drug testing represents an application where system scalability is particularly attractive. Therefore, we tested our systems on tissue exposed to a well-characterized and clinically relevant calcium channel blocker, nifedipine, which has been used to treat angina and hypertension. As experimental models, we used the Langendorff-perfused whole-heart and thin ventricular tissue slices, a preparation gaining renewed interest by the cardiac research community. Using our simplified systems, we were able to monitor simultaneously the marked changes in the voltage and calcium transients that are responsible for the negative inotropic effect of the compound.

**Electronic supplementary material:**

The online version of this article (doi:10.1007/s00424-012-1149-0) contains supplementary material, which is available to authorized users.

## Introduction

In vitro testing of drug-induced effects on cardiac electrophysiology is important for both academic and pharmaceutical research [45]. From a basic science standpoint, drug interventions allow one to perturb biochemical pathways, and this can lead to insights into mechanisms underlying pathophysiological behaviour. From a pharmaceutical standpoint, rapidly obtained data both on target mechanisms and cardiac side effects are critical for the assessment of efficacy and safety prior to embarking on expensive in vivo drug testing. Much of our understanding of cardiac electrophysiology has relied on the Langendorff-perfused isolated mammalian heart model, developed over 100 years ago [44]. Key parameters of interest in cardiac electrophysiology research are the trans-membrane voltage (*V*_m_) and resulting intracellular calcium concentration ([Ca^2+^]_i_) changes which regulate heart contraction [3]. Using Langendorff heart preparations in combination with fluorescent dyes and sophisticated high-speed imaging instrumentation, one can study *V*_m_ and [Ca^2+^]_i_ signals at both the cellular level and the level of macroscopic wave dynamics. Understanding this complex physiology is crucial to the study of cardiac pathophysiology and drug mechanisms of both cardiac and non-cardiac medications. Hence, simultaneous imaging of *V*_m_ changes associated with the cardiac action potential (AP) and of the intracellular calcium transient (CaT) responsible calcium-induced calcium release (CICR) using fluorescence methods has become an indispensable tool [14, 32, 40].

Despite its importance to the field, optical mapping of whole-heart preparations remains a complex and expensive procedure, requiring significant expertise. This has limited its application and the range of experiments possible. In terms of instrumentation, the limiting factors relate to the sensor (usually a camera) and the light source, not only because they are the two most important components but also because they are often very expensive. The cameras most widely used by the community tend to cost over £20,000 [16]. Even though equipment costs should not be a limitation of the scientific process, in reality, the financial burden, especially if multiple detectors are required, can be a make-or-break issue. For multiple parameter observation, the default approach utilises two cameras (and a dichroic beam splitter to project separate fluorescence emissions on the two detectors). This approach has provided much insight into *V*_m_ and [Ca^2+^]_i_ dynamics under normal conditions and in disease [11, 21, 29, 30, 34]. Although some applications require such high-performance systems, other questions can be addressed at lower spatial and temporal resolution. For the latter, we see an area of high demand for alternative imaging solutions that offer scalability through technological simplicity.

Fortunately, advances both in illumination and camera technology allow realization of this goal. In terms of illumination, traditional light sources (such as xenon/halogen/mercury lamps combined with suitable excitation filters) can be replaced by powerful light-emitting diodes (LEDs) [1, 17]. Not only are LEDs affordable alternatives that offer stable light output intensities but also their output can be adjusted at very high speeds (response times in the nanosecond to microsecond range). On the imaging side, advances in camera technology have provided alternatives to expensive scientific speciality devices. In particular, camera speeds have increased to the point where multi-colour imaging using a single camera is now possible, significantly decreasing technical complexity (such as by avoiding the need for detector co-alignment [47]). This multi-colour technique has recently been applied to cardiac optical mapping [31]. Single-camera recording of multiple parameters using similar alternation of excitation sources has also been recently reported [2, 42]. Moreover, while optical mapping techniques are important to some applications, even simpler optical fibre-based fluorescence detection of the heart is feasible when only one or few points on the heart need to be probed (e.g. for exploring the effects of a drug on the AP and CaT by comparing control/intervention/wash-out), eliminating the need for complex image processing. Much work has been devoted to optical fibre-based detection, including measuring *V*_m_ alone [4, 6, 8, 22, 24, 25, 28, 37], CaT alone [10, 36], *V*_m_ and CaT separately [27, 46], and *V*_m_ and CaT simultaneously [26]. Most of these designs utilize traditional light sources (laser, mercury/arc lamp) and detectors (photomultiplier tube, avalanche photodiode and photodiode array). Although a multi-parametric detection system has been reported [26], this design used an expensive spectrograph and linear photodiode array system. Surprisingly, a simple simultaneous *V*_m_ and CaT optical fibre-based system which provides high temporal resolution has not yet been established.

In addition to instrumentation technology, recent developments in cardiac preparations offer new opportunities for more refined model systems. Cardiac tissue slices [12, 39] have gained a renewed interest by the cardiac experimental and modelling communities [13] because they represent a pseudo-2D model (thickness is between one to two orders of magnitude lower than the in-plane dimensions), which avoids some of the pitfalls of whole-heart fluorescence studies (e.g. photon scattering depth-effects) [5, 13]. Inside the slices, cells are exposed to electrophysiological source–sink and local environmental conditions that are more similar to the native state than in cell cultures, a widely used 2D experimental model. In contrast to the widespread use of brain slices in neuroscience, cardiac tissue slices have received comparably little attention for many years, with only a few 2D optical electrophysiological studies performed [12, 39]. Owing to the delicate nature of the slice preparation, injury from cutting must be minimized. With improvements in vibratome technology that feature low blade advancement speeds (~mm/min) and sub-micrometer out-of-plane blade deviations, tissue slices suitable for experimentation can be prepared. As cardiac tissue slices can be cut as thinly as 250–350 μm (to avoid severe restriction of oxygen diffusion), it is possible to harvest multiple slices from a single heart, even if cut in the tangential plane to optimise cell preservation, permitting a thorough investigation of the myocardium. In the context of drug testing, cardiac tissue slices offer a very interesting model system [7, 9]. For example, a simple optical fibre-based system (described below) could be scaled to probe many tissue slices in parallel, opening the door to high-throughput drug testing in native tissue. Because multiple slices from the same heart may be exposed systematically to a range of compounds, a large volume of data can be generated with reduced inter-individual variability.

In this paper, we describe two systems: System 1 is camera-based and allows *V*_m_ and CaT mapping using the multi-colour imaging technique with an ‘economy’ electron-multiplying charge-coupled device (EMCCD) camera (~£7,800). In addition, as a proof-of-principle, we demonstrate CaT mapping with a high-speed consumer camera and LEDs. Although such cameras cannot replace EMCCD cameras for demanding scientific applications, they may be sufficient in high light-level physiological and educational studies, e.g. for larger rodent Langendorff heart preparations. System 2 is an optical fibre-based *V*_m_ and CaT detection system using one LED and two off-the-shelf single-element photodiodes (~£40 each). To test the applicability of our system for studying cardiac drug effects, we exposed the cardiac tissue to nifedipine, a 1,4-dihydropyridine calcium channel blocker (DCCB) [23]. This also serves as an illustration of pharmacological side-effect profiling, as micromolar-level concentrations of nifedipine have been shown to substantially reduce AP duration and CaT amplitude (associated with reduced influx of calcium into the cell leading to reduced calcium ion release from the sarcoplasmic reticulum) [18, 20, 33, 43].

## Methods

### Isolated Langendorff-perfused guinea pig whole-heart

Hearts were isolated from female guinea pigs (300–500 g) after cervical dislocation and in accordance with Schedule 1 of the UK Home Office Animals (Scientific Procedures) Act of 1986, and swiftly connected to a Langendorff perfusion setup [*n* = 3 for camera-based multi-parametric whole-heart imaging; *n* = 2 for camera-based multi-parametric tissue-slice imaging (at least four tissue slices per left ventricle); *n* = 2 for consumer camera-based whole-heart imaging; *n* = 3 for optical fibre-based detection]. Hearts were perfused at a constant rate of 8 mL/min with a modified Krebs–Henseleit solution (containing, in millimoles per litre: NaCl 125, CaCl_2_ 1.8, KCl 5.4, NaHCO_3_ 25, NaH_2_PO_4_∙H_2_O 1.2, MgCl_2_ 1, glucose 5.5, probenecid 0.2; all from Sigma-Aldrich, Dorset, UK) gassed with 95 % O_2_/5 % CO_2_ to maintain a pH of 7.4.

Fluorescent dyes were injected into the aortic cannula for coronary perfusion. For CaT imaging, hearts were dye-loaded by re-circulating 100 mL perfusate containing 2.5 μmol/L fura-4F AM (Life Technologies, Paisley, UK) or rhod-2 AM (Life Technologies) for 30 min. For *V*_m_ imaging, the heart was loaded with dye by delivering, without recirculation, a 20-μL bolus of 27.3 mmol/L (in pure ethanol) di-4-ANBDQPQ (Richard D. Berlin Center for Cell Analysis and Modeling, University of Connecticut Health Center, USA), applied slowly over 5 min (i.e. diluted in 40 mL perfusate), or a 20-μL bolus of 15 μmol/L (in DMSO) RH237 (Life Technologies), also applied over 5 min (i.e. diluted in 40 mL perfusate). To load di-4-ANBDQPQ, Pluronic F-127 (Life Technologies) was added to the bolus, to a final concentration of 0.2–0.5 % [35].

For suppression of motion, excitation–contraction was uncoupled with blebbistatin (Sigma-Aldrich) at 10 μmol/L [19]. All whole-heart experiments were conducted at 36 ± 1 °C. Nifedipine (N7634; Sigma-Aldrich) from a 20-mmol/L stock solution (dissolved in DMSO) was diluted in the perfusate to 2 μmol/L.

### Guinea pig left ventricular tissue slices

For tissue slices, hearts were isolated and loaded with dye as described above. After suppressing contractile activation with 2,3-butanedione 2-monoxime (BDM, 10 mmol/L; Sigma-Aldrich), we commenced the tissue slicing procedure, using a technique described elsewhere [7]. Briefly, a tissue chunk of the left ventricle was glued with Histoacryl tissue adhesive (Aesculap AG & Co. KG, Tuttlingen, Germany) onto a 4 % agarose gel block (NuSieve GTG Agarose; Lonza, Slough, UK) that was fixed on top of the vibratome cutting stage (Campden Instruments Ltd., Loughborough, UK). Slices in the tangential plane relative to the epicardial surface (350 μm thick) were cut with a steel blade at a progression speed of 0.03 mm/s, amplitude 2 mm, and vibration frequency 80 Hz. During cutting, the tissue slice was submerged in ice-cold (0 °C) BDM containing Tyrode's solution (in millimoles per litre: NaCl 140, KCl 5.4, MgCl_2_ 1, HEPES 5, glucose 11, CaCl_2_ 1.8, BDM 10; bubbled with O_2_). Cut slices were transferred to a custom imaging chamber and pinned down using insect preparation pins onto a polydimethylsiloxane block in order to prevent curling. Tissue slices were imaged in blebbistatin-containing modified Krebs–Henseleit solution. Nifedipine was added from a 20-mmol/L stock solution (dissolved in DMSO) and diluted in the chamber solution to a final concentration of 2 μmol/L.

### Camera-based multi-parametric imaging

**Fig. 1:**
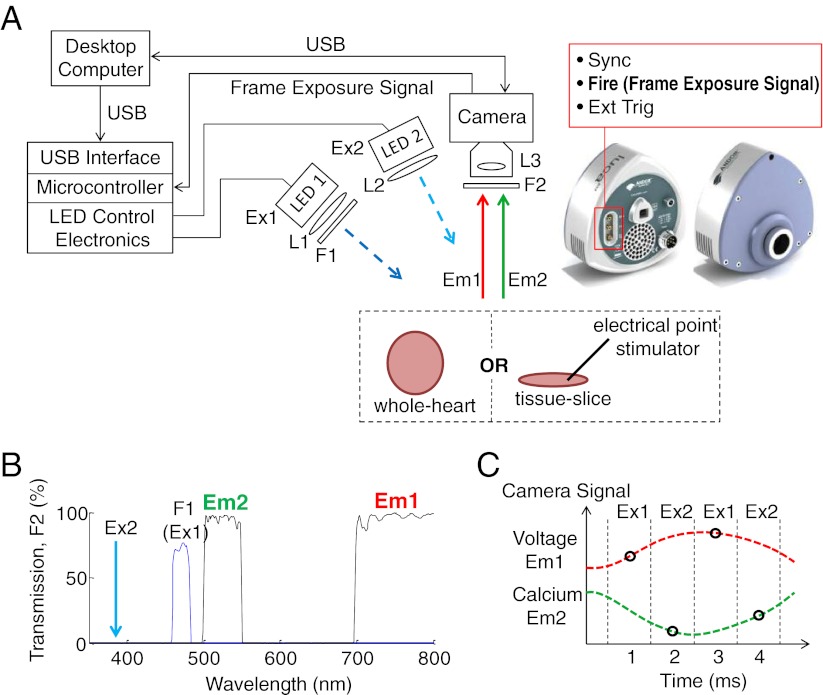
Schematic of EMCCD camera-based whole heart/tissue slice trans-membrane voltage (*V*_m_) and intracellular free calcium concentration ([Ca^2+^]_i_) dynamics optical mapping system. **a** System layout with key components (see text for detail). Two pictures showing the front and back of the compact USB camera are shown. **b** The transmission spectra of the multi-band emission filter passing both *V*_m_ (Em1) and [Ca^2+^]_i_ (Em2) dye emission. The excitation filter, *F1*, for the *V*_m_ dye excitation source, *Ex1*, is shown. The ultraviolet (385-nm centred) excitation source for the [Ca^2+^]_i_ dye is also represented (no excitation filter required). **c** The single-sensor multi-colour imaging technique: During any frame exposure, the *V*_m_/[Ca^2+^]_i_ (*Em1*/*Em2*) signal is acquired by illuminating the tissue with excitation source *Ex1*/*Ex2*, respectively. At high-speed and with interpolation (see dotted curves), this method provides a straightforward method of simultaneously imaging two parameters

The system implemented here is a modification of a four-parameter imaging system described elsewhere [31], where detailed circuit diagrams and software code are provided. Primary differences lie in the cameras used and the custom multi-band emission filter. Provided below is a brief description of the setup (Fig. 1).

Excitation of di-4-ANBDQPQ is done using the following:

- LED1: CBT-90-B (peak power output 53 W; peak wavelength 460 nm; Luminus Devices, Billerica, MA, USA)
- L1: plano-convex lens (LA1951; focal length = 25.4 mm; Thorlabs, Ely, UK)
- F1: D470/20X (Chroma Technology, Bellows Falls, VT, USA)

Excitation of fura-4F AM is done using the following:

- LED2: NCSU034A (peak power output 400 mW; peak wavelength 385 nm; Nichia, Tokushima, Japan)
- L2: plano-convex lens (LA1951; focal length = 25.4 mm; Thorlabs)

Fluorescence emission from whole hearts/tissue slices is passed through a custom (now off-the-shelf) multi-band emission filter F2: ET585/50-800/200 M (Chroma Technology) and collected with a ‘fast’ camera suitable lens (f/# 0.95; DO-1795; effective focal length = 17 mm; Navitar, Rochester, NY, USA). Fluorescence images are taken with a high-speed EMCCD camera (Luca(S); Andor Technology, Belfast, Northern Ireland) set at (a) 125 × 75 effective super pixels (4 × 4 binning) for the whole heart and (b) 125 × 77 effective super pixels (2 × 2 binning) for tissue slices. At these pixel resolutions (i.e. 125 × 75 and 125 × 77), the camera acquires images at 195 frames-per-second (fps). Binning, 4 × 4, permitted the projection of the larger whole heart fluorescence image onto a larger camera sensor area, while 2 × 2 binning permitted the projection of the smaller tissue-slice fluorescence image onto a smaller camera sensor area. Andor Technology's Solis software, which is provided with the camera, was used to configure the camera and to acquire images.

The microcontroller-based interface synchronises excitation light switching with EMCCD camera frame exposures (*Fire* signal from camera rear). The LEDs are controlled with a custom-built high-power LED driver circuit (for details, please refer to [31]). An eight-processor microcontroller (Propeller chip; Parallax, Rocklin, CA, USA) is used to control and coordinate all major components of the setup. Software for time-critical tasks was written in the microcontroller's assembly language. The Andor camera has a shorter transfer time between frame exposures (a few microseconds) compared to the camera used in previous work [31]. Accordingly, to ensure lack of bleed-through into the next frame, the LED light sources are turned off before the end of the frame exposure (when set to high power, LED off-times can exceed 5 μs). Communication with a standard desktop computer is achieved with a USB interface module (UM245R; Future Technology Devices International, Glasgow, UK). Custom software written in MATLAB (MathWorks, Natick, MA, USA) was used to communicate with the microcontroller and perform optical mapping image processing. All electronic components were acquired from major electronic components distributors (e.g. Digi-Key Corp., Thief River Falls, MN, USA).

Whole hearts were imaged in sinus rhythm; tissue slices were electrically stimulated at 2 Hz with biphasic pulses having an amplitude of ~5 V and duration of 3 ms, generated by a custom-built stimulator. A bipolar concentric stimulation electrode was used (Lohmann Research Equipment, Castrop-Rauxel, Germany).

### Consumer camera-based whole-heart optical mapping

The heart was illuminated with a filtered green LED light source (Fig. 4a):

- LED: CBT-90-G (peak power output 58 W; peak wavelength 524 nm; Luminus Devices)
- L1: plano-convex lens (LA1951; focal length = 25.4 mm; Thorlabs)
- F1: green excitation filter (D535/25X; Chroma Technology)

An EX-FH100 high-speed digital camera (Casio Computer Co., Tokyo, Japan), with an emission filter (ET585/40 M; Chroma Technology) suitable for rhod-2 placed in front of the lens, was used to optically map CaT in the whole heart (Fig. 4). The camera was manually focused with 1/250 s exposure at 240 fps (448 × 336 pixels), with a sensitivity setting of ≥ ISO 1,600. AVI movie files were converted into a matrix stack (3D matrix, horizontal pixels × vertical pixels × time) in MATLAB then analyzed. See below for sample MATLAB code to read EX-FH100 generated AVI files. R, G and B frames were summed up into one representative frame because transmission spectra of R, G and B components of the Bayer filter all overlap with the calcium dye (rhod-2) emission spectrum.

MATLAB code to read in AVI movie files into a 3D matrix (i.e. a stack of image frames) for the Casio EX-FH100 high-speed digital camera:
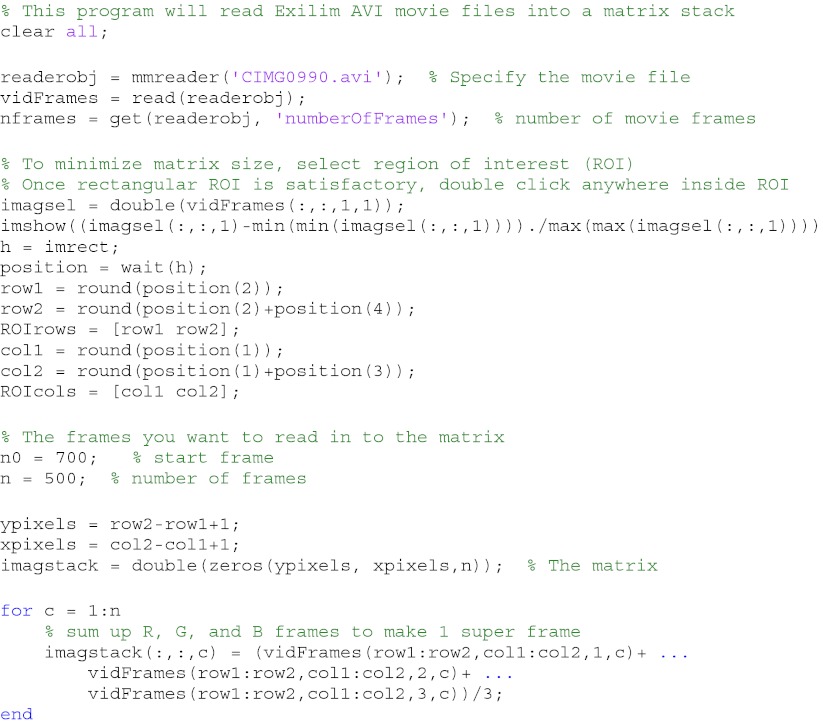


### Optical fibre-based multi-parametric detection

In Fig. 5, a 5 mm core liquid light guide was used (77636; Newport Corporation, Didcot, UK). We experimented also with a 1-mm diameter multi-mode optical fibre (M35; Thorlabs) as an alternative to the light guide. The smaller 1-mm diameter fibre required more amplifier gain (i.e. comparatively less fluorescence emission collection), but gave similar signal quality (data not shown).

The main body of the backend is composed of two sets of cage cubes: (1) 30-mm cage system cube (C6W), (2) rotatable cage cube platform (B3C) and (3) cage-compatibledichroic filter mount (FFM1). Dichroics D1 and D2 are mounted in this main body, and the fibre, excitation source and detectors are attached at the periphery (Fig. 5). All parts are from Thorlabs' 30-mm cage components. The following is a list of the key components (refer to Fig. 5):

- L1, L3, L4: achromatic doublet lens (AC254-030-A-ML; focal length = 30 mm; Thorlabs)
- L2: plano-convex lens (LA1951; focal length = 25.4 mm; Thorlabs)
- F1: green excitation filter (D535/25X; Chroma Technology)
- D1: 25.5 × 36 mm dichroic beam splitter (565DCXR; Chroma Technology)
- D2: 25.2 × 35.6 mm dichroic beam splitter (FF705-Di01-25x36; Semrock, Rochester, NY, USA)
- F2: Rhod-2 emission filter (ET585/40 M; Chroma Technology)
- PD1, PD2: silicon single-element photodiode with ultraviolet enhanced response (NT57-510; Edmund Optics, York, UK)
- LED: CBT-90-G (peak power output 58 W; peak wavelength 524 nm; Luminus Devices)

A circuit diagram of the photodiode amplifier electronics and LED driver can be found in Fig. 6 (the LED and power transistor require heat-sinking to ensure LED output power stability). Amplifier *V*_m_ outputs were captured on a digital oscilloscope (PicoScope; Pico Technology, Cambridge, UK). Sample results from higher bandwidth amplifier electronics with the low-pass filter removed are shown in Fig. 7.

### Image processing

Custom software written in MATLAB was used to perform optical mapping image processing. Although time-course signals were left unfiltered in time, images for normalized fluorescence intensity maps and movies were filtered using 2D median filtering (MATLAB's built-in *medfilt2* function). All *V*_m_ and [Ca^2+^]_i_ transient signals shown are presented unfiltered in time to demonstrate the sufficient signal quality achieved with the much cheaper technology presented (it should be noted, though, that consumer camera systems typically apply smoothing algorithms not easily changeable by end-users).

## Results and discussion

### Camera-based multi-parametric imaging

A schematic of the camera-based *V*_m_ and CaT optical mapping system is shown in Fig. 1a. The *V*_m_ and [Ca^2+^]_i_ dyes used were di-4-ANBDQPQ and fura-4F AM, respectively. Di-4-ANBDQPQ is a second-generation ratiometric *V*_m_ dye that is typically excited in the blue and red (preferred for larger signal swings) wavelength ranges [35]. Fura-4F AM is a low-affinity version of the widely used ratiometric [Ca^2+^]_i_ dye, fura-2. Although fluorescence signals are significantly smaller using low-affinity dyes, the benefit of a lower affinity is that it both minimally perturbs [Ca^2+^]_i_ dynamics on one hand, and more accurately tracks fast [Ca^2+^]_i_ signal kinetics at peak concentrations, such as involved in CICR [48]. The lack of cross-talk between di-4-ANBDQPQ and fura dyes has been established previously [31]. Di-4-ANBDQPQ is excited with a filtered blue-LED (Ex1), and fura-4F AM is excited with a narrow-band ultraviolet LED (385-nm centred; Ex2). Spherical lenses are used to collimate both excitation sources. A blue light source, instead of red, is chosen for di-4-ANBDQPQ to have more similar wavelength excitation sources, leading to more similar tissue penetration depths [5]. Ex1 produces *V*_m_ signals in the longpass band (Em1), and Ex2 produces [Ca^2+^]_i_ signals in the pass-band (Em2) of the multi-band filter (Fig. 1b). Using the multi-colour imaging technique, during any camera frame exposure, only Ex1 or Ex2 is turned on [31]. Hence, the emitted fluorescence signal during any frame exposure represents either the *V*_m_ or CaT signal, respectively (Fig. 1c). If the camera is sufficiently fast (~200 fps in the system described here) compared to the signal dynamics, with interpolation, accurate simultaneous time courses of both signals can be measured. Admittedly, this frame rate will not be sufficient for all applications (e.g. characterizing the upstroke dynamics of the AP), but it is suitable for characterizing AP durations and shapes, or rotors during ventricular fibrillation [41]. Blebbistatin, a commonly used contraction uncoupler, was used to image cardiac tissue electrical activity without motion-causing imaging ‘artefacts’ [19].

We decided to test this system in control conditions and in a whole heart exposed to a pharmacologic agent known to cause changes in the cardiac AP and CaT. Nifedipine was chosen because it was found to have cardiotoxic side effect, which has led to significant restrictions regarding its use in patients. Like other DCCBs, nifedipine is known to decrease cardiac CICR. This effect was found to have profound negative-inotropic consequences when used in patients with cardiomyopathy as documented in a randomized clinical trial where nifedipine use, while hypothesized to be beneficial in cardiomyopathy for its afterload-reducing properties, was found to exacerbate heart failure hospitalizations [15]. As a result, the use of DCCBs is now contraindicated in patients with cardiomyopathy, being replaced by newer non-DCCBs developed to avoid the aforementioned cardiotoxic side effects [38]. Therefore, a simple and scalable tool that could have screened for this effect might have proved useful at the time of drug discovery.

**Fig. 2:**
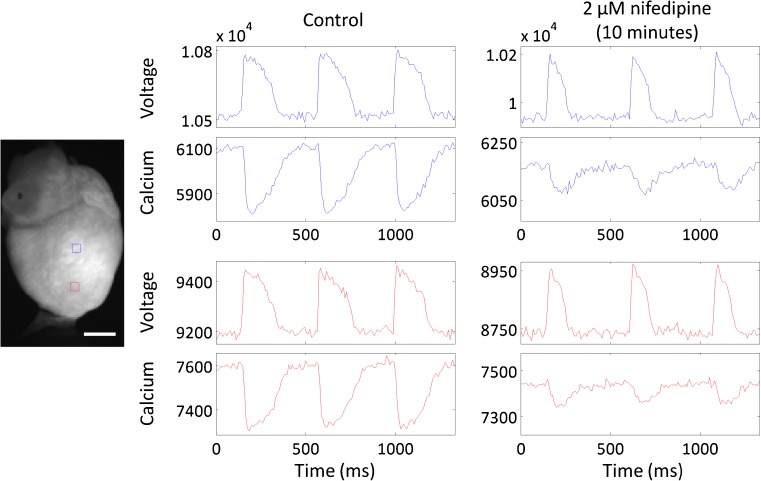
Whole-heart (mostly left ventricle in view; guinea pig) optical mapping of *V*_m_ and CaT. Raw (unfiltered in time) *V*_m_ and CaT signals, on a 14-bit scale, are shown from two regions (6 × 6 pixels) of the heart in sinus rhythm. The *left column* shows the control signals before drug application, and the *right column* shows the altered signals after 10 min of exposure to 2 μM nifedipine. Note reduction in heart rate, AP duration and CaT amplitude. *Scale bar* = 5 mm

The camera-based multi-parametric imaging system described in Fig. 1 was applied to both the guinea pig whole heart and left ventricular tissue slices exposed to 2 μM nifedipine. Figure 2 shows sample *V*_m_ and CaT changes from two points on the ventricle of the heart under control conditions and after nifedipine administration. As expected, in response to nifedipine, there is a notable reduction in heart rate, AP duration and CaT amplitude (~69 % reduction in Δ*F*/*F* for the heart shown). A movie of sinus rhythm activation before application of nifedipine can be found in Supplementary Movie 1.

**Fig. 3:**
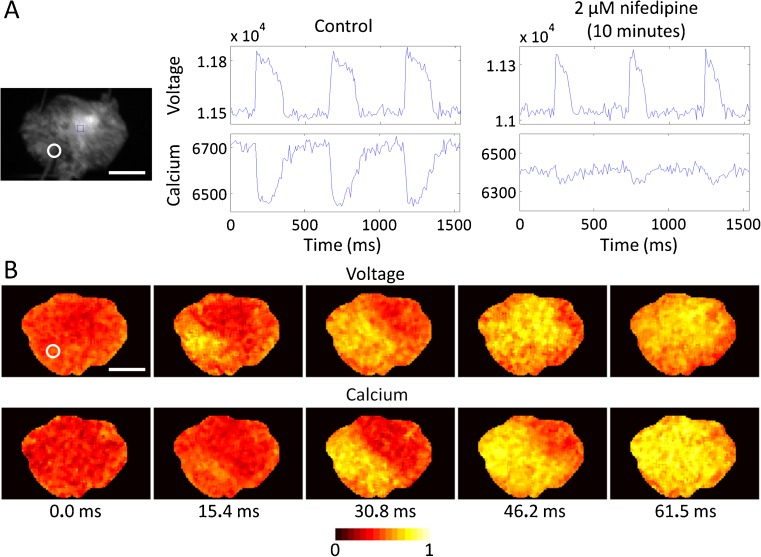
Tissue-slice (350 μm thick; guinea pig) optical mapping of *V*_m_ and CaT. **a** Raw (unfiltered in time) *V*_m_ and CaT signals, on a 14-bit scale, are shown from a region (6 × 6 pixels) of the tissue slice during 2 Hz stimulation (at the site of the *white circle*). The *left column* shows the control signals before drug application, and the *right column* shows the altered signals after 10 min of exposure to 2 μM nifedipine. Note reduction in AP duration and CaT amplitude. **b** Normalized fluorescence intensity maps (*colour bar* shown) of *V*_m_ and CaT at five time points, showing the progression of the activation wave. *Scale bar* = 5 mm

Figure 3 shows sample results from a tissue-slice preparation under the same conditions as in Fig. 2 (also control and after nifedipine administration). The tissue slice was electrically stimulated at 2 Hz. Similar to whole-heart experiments, a reduction in AP duration and CaT amplitude (~74 % reduction in Δ*F*/*F* for the tissue slice shown) was observed after 10 min of nifedipine exposure (Fig. 3a). Figure 3b shows normalized fluorescence intensity maps from corresponding *V*_m_ and intracellular CaT (upper and lower panels, respectively) at five progressive time points (see Supplementary Movie 2) from the same tissue slice as in Fig. 3a under control conditions. The data illustrate the excitation wave progressing into the tissue from the stimulation site, and the well-known delay in CaT (by comparison to the *V*_m_ signal). For both Figs. 2 and 3a, signals are unfiltered in time to demonstrate the quality afforded by the EMCCD camera used.

### Consumer camera-based whole-heart optical mapping

**Fig. 4:**
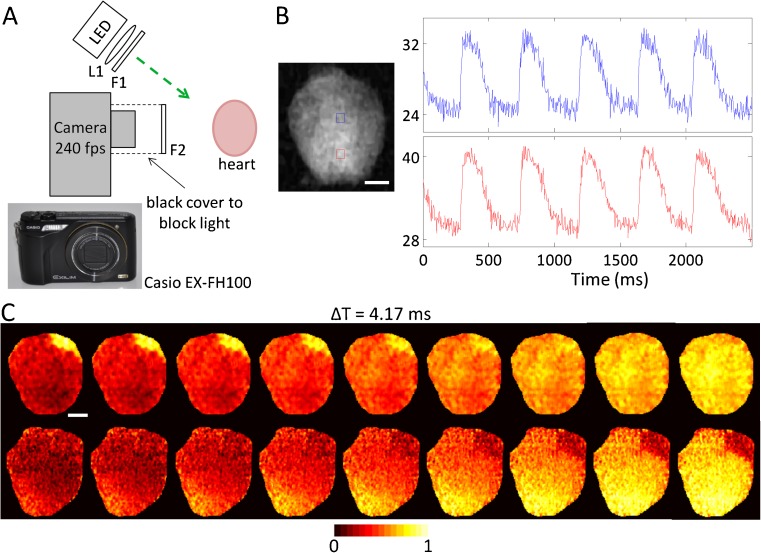
Schematic and sample results from a high-speed consumer camera-based whole-heart CaT imaging system. **a** Whole-heart CaT optical mapping setup. A green LED excitation source is collimated (lens *L1*), filtered (*F1*) and targeted towards the heart. An emission filter (*F2*) for the rhod-2 dye is placed in front of the camera lens and a black cover used to eliminate stray light. A picture of the Casio EX-FH100 is shown. **b** Raw (unfiltered in time) CaT signals, on an 8-bit scale, are shown from two regions (10 × 10 pixels) of the heart (mostly left ventricle view) in sinus rhythm. **c** Normalized fluorescence intensity maps (*colour bar* shown) of CaT at nine sequential time points, showing the progression of whole-heart [Ca^2+^]_i_ changes in sinus rhythm (*top panel*). The *bottom panel* shows another heart being paced at the apex at 5 Hz. *Scale bar* = 5 mm

Advances in high-speed consumer cameras that enable video recording rates up to 1,000 fps open up many intriguing opportunities for economical experimental studies of high-speed biological phenomena. The ability to read data (frame-by-frame) into a numerical computing environment like MATLAB breaks down some barriers to data access for computational biologists. To evaluate the sensitivity of the sensor and the suitability of such cameras for high light-level optical mapping and educational applications, we used the EX-FH100, a unit from Casio's Exilim line of high-speed digital cameras (Casio Computer Co.). This camera is based on a sensitive back-illuminated complementary metal oxide semiconductor (CMOS) sensor. Because of the Bayer filter in front of the sensor, we used the rhod-2 dye, which emits in the red filter range. A schematic of the whole-heart CaT optical mapping setup is shown in Fig. 4a. A filtered green LED excitation source was used to illuminate the rhod-2 loaded heart, and fluorescence light is collected through an emission filter (F2). Figure 4b shows sample CaT signals from two regions on the left ventricle of a guinea pig whole heart, and Fig. 4c shows normalized fluorescence intensity maps at nine sequential time points to illustrate whole-heart CaT wave spread in sinus rhythm (top panel; see Supplementary Movie 3; Supplementary Movie 4 shows a 30-fps playback of the sequence as saved on the camera, before processing). The bottom panel of Fig. 4c shows normalized fluorescence intensity maps of another heart being paced at the apex at 5 Hz (see Supplementary Movie 5; Supplementary Movie 6 shows a 30-fps playback of the sequence as saved on the camera, before processing). Although difficult with some consumer camera systems, replacing the given camera lens with a ‘fast’ imaging-suitable lens (not only improving fluorescence emission collection but also maximizing pixel use) should improve sensitivity and signal quality. In addition, consumer camera systems apply complex image processing algorithms, generating data that is not raw and limiting interpretability. Until more flexibility is afforded to the user, the range of high-speed biological imaging applications will be limited. It should also be noted that consumer cameras are unlikely to replace EMCCD cameras, but they can be considered in certain high light-level fluorescence and educational applications e.g. university classroom.

### Optical fibre-based multi-parametric detection

**Fig. 5:**
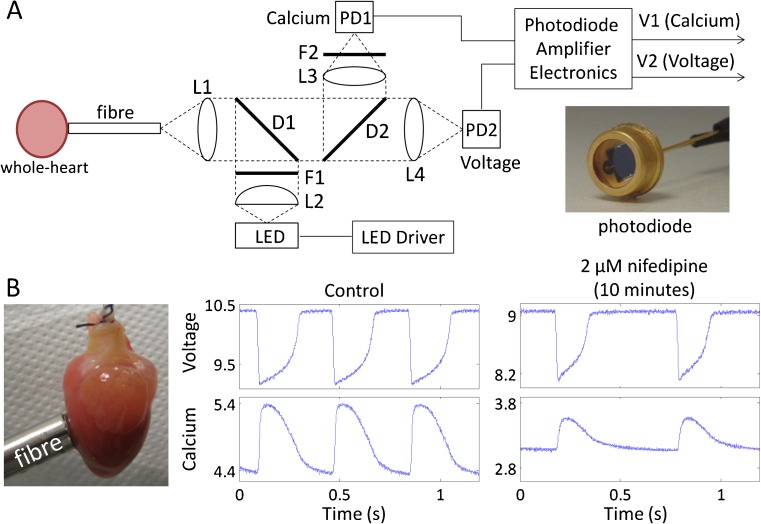
Schematic and sample results from an optical fibre-based *V*_m_ and CaT detection system. **a** Schematic of the optical fibre system backend (fibre-proximal end). *L1*, *L2*, *L3* and *L4* are collimating lenses; *D1* is a dichroic beam splitter used to separate excitation and emission light; *D2* is a dichroic beam splitter used to separate *V*_m_ and CaT emission light; *F1* is an excitation filter; *F2* is a CaT emission filter; *PD1* and *PD2* are photodiodes used to detect CaT and *V*_m_ emission, respectively; *V1* and *V2* are amplifier output signals representing CaT and *V*_m_ emission, respectively. A picture of the photodiode used is shown. **b** *V*_m_ and CaT signals (obtained using a digital oscilloscope) are shown from a region of the heart's left ventricle in sinus rhythm. The *left column* shows the control signals before drug application, and the *right column* shows the altered signals after 10 min of exposure to 2 μM nifedipine. Note reduction in heart rate, AP duration and [Ca^2+^]_i_ transient amplitude. The *left panel* shows a photograph of the fibre's distal end, resting gently against the left ventricle of a guinea pig heart

**Fig. 6:**
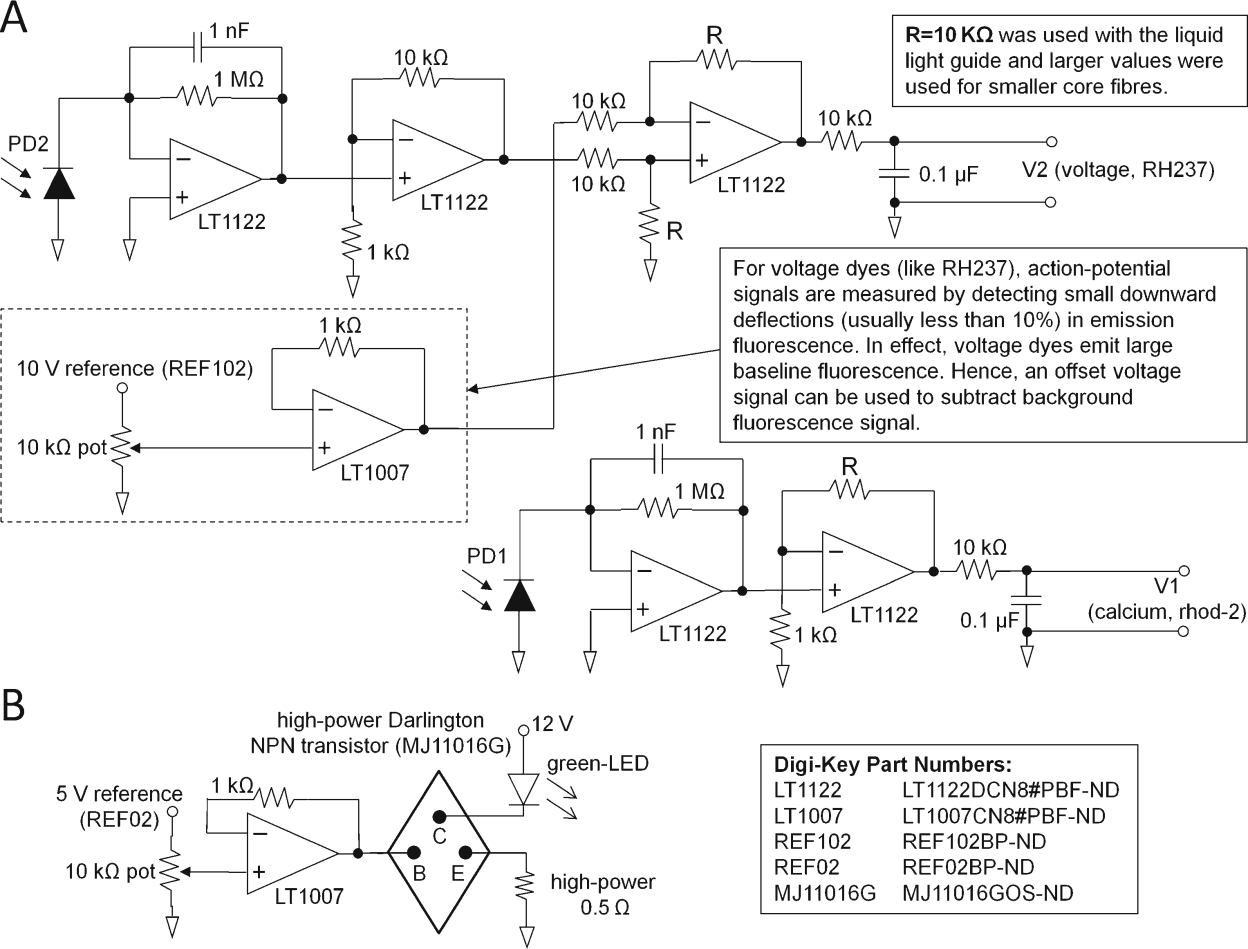
Photodiode amplifier electronics and LED drive electronics. **a** A schematic of the photodiode amplifier circuit used to measure *V*_m_ and [Ca^2+^]_i_ fluorescence (Fig. 5). **b** A schematic of the green LED drive circuit (used for both the optical fibre system and the consumer digital camera system). Part numbers for key components are also shown (Digi-Key Corp., Thief River Falls, MN, USA)

**Fig. 7:**
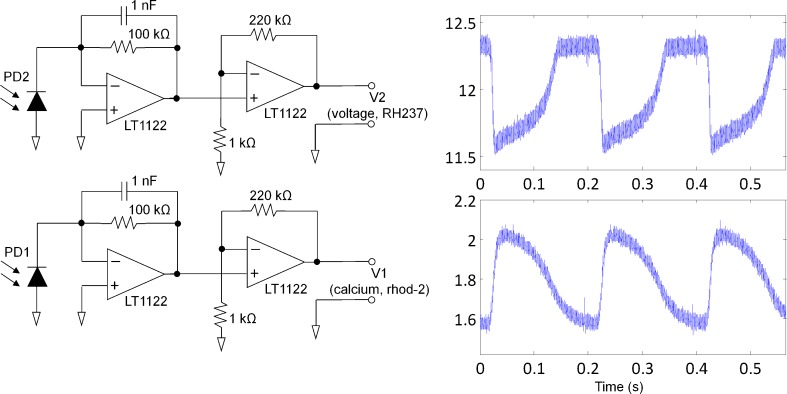
Higher bandwidth amplifier electronics and sample results. A schematic of a higher bandwidth amplifier circuit, without low-pass filter, is shown on the *left*. Sample results are shown on the *right* (*top panel*: *V*_m_, *bottom panel*: [Ca^2+^]_i_), acquired from a digital oscilloscope sampling at 19.61 KHz

While for optical mapping it is important to obtain spatially resolved representation of cardiac electrophysiology and ion changes, data recorded from a single point on the heart surface can be sufficient for before–after comparisons. High quality instrumentation for *V*_m_ and CaT measurements is therefore desirable. Here, we chose to implement such a system for *V*_m_ and CaT using an optical fibre-based probe, which is shown in Fig. 5. We used RH237 (for *V*_m_) and rhod-2 (for [Ca^2+^]_i_), not only because it is probably the most widely used dye combination in the cardiac multi-parametric imaging community [11, 29, 34] but also to simplify the design by using a single light source for simultaneous *V*_m_ and [Ca^2+^]_i_ dye excitation. To our knowledge, optical fibre-based detection, using single-element photodiodes, to observe RH237 and rhod-2 dynamics has not been reported. The backend of the system is shown in Fig. 5a. A filtered (F1) green LED provides excitation light into the fibre's proximal end by reflecting off dichroic D1. RH237 and rhod-2 emission light from the proximal end is passed through dichroic D1 and then gets separated by dichroic D2. With additional emission filtering (F2), rhod-2 fluorescence is collected with photodiode PD1, and RH237 fluorescence is collected with photodiode PD2. The photodiode amplifier electronics (Fig. 6) uses off-the-shelf components (Digi-Key Corp.) and can easily be prototyped on a breadboard (i.e. all chips are available in DIP package format). Figure 5b shows sample *V*_m_ and CaT signals from a region on the left ventricle of a guinea pig whole heart, measured with this optical fibre-based system, generating high signal-to-noise ratios with the prototypical response described above to nifedipine (results from a higher bandwidth amplifier circuit can be found in Fig. 7). Because the fibre's distal end was pushed gently against the ventricle, the use of blebbistatin could be avoided.

## Conclusion

In this paper, we have shown that recent advances in high-speed cameras, photodiodes, multi-band optical filters and powerful light-emitting diodes make it possible to construct high quality, multi-parametric electrophysiological imaging systems, needed for high-throughput applications such as drug testing. We provide a proof-of-principle application, using a known pharmaceutical compound, to demonstrate that its potentially cardiotoxic side effect can be detected with the described systems. As further technological advances are made, cardiac imaging tools will continue to be developed with the potential to greatly increase the yield for this important scientific and clinical tool.

## Electronic supplementary material

Below is the link to the electronic supplementary material.

Supplementary Movie 1 Whole-heart (guinea pig) trans-membrane voltage (*V*_m_) and intracellular free calcium concentration ([Ca^2+^]_i_) dynamics in sinus rhythm (prior to nifedipine application) using the ‘economy’ EMCCD camera-based optical mapping system (Fig. 1) (MPEG 4510 kb)

Supplementary Movie 2 Tissue-slice (guinea pig left ventricle) *V*_m_ and CaT, activation after electrical point stimulation (prior to nifedipine application) using the ‘economy’ EMCCD camera-based optical mapping system (Fig. 1) (MPEG 2844 kb)

Supplementary Movie 3 Whole-heart (guinea pig) activation wave of CaT in sinus rhythm using the Casio EX-FH100 high-speed digital camera (Fig. 4) (MPEG 6332 kb)

Supplementary Movie 4 Playback movie of the same heart at 30 fps (camera was run at 240 fps) before processing in MATLAB. In effect, it is the movie as is saved on the camera (MPEG 1130 kb)

Supplementary Movie 5 Whole-heart (guinea pig) activation wave of CaT during 5 Hz apex pacing using the Casio EX-FH100 high-speed digital camera (Fig. 4) (MPEG 5028 kb)

Supplementary Movie 6 Playback movie of the same heart at 30 fps (camera was run at 240 fps) before processing in MATLAB. In effect, it is the movie as is saved on the camera (MPEG 1314 kb)
